# Anionic polyelectrolytes in titanosilicate molecular sieve synthesis towards simultaneously accomplishing low production cost and high catalytic activity†

**DOI:** 10.1039/c8ra02621a

**Published:** 2018-06-13

**Authors:** Kairui Fu, Jingui Wang, Yichen Wang, Yuanchao Shao, Jiaqi Zhu, Tianduo Li

**Affiliations:** Shandong Provincial Key Laboratory of Fine Chemicals, School of Chemistry and Pharmaceutical Engineering, Qilu University of Technology (Shandong Academy of Sciences) Jinan 250353 P. R. China JGWang@qlu.edu.cn ylpt6296@vip.163.com

## Abstract

Assisted by an anionic polyelectrolyte of poly(acrylic acid) (PAA), high-performance titanium silicalite-1 (TS-1) could be facilely synthesized at very low usage of expensive organic templates (tetrapropylammonium hydroxide). The presence of PAA helped to incorporate more active Ti species into the TS-1 framework and change the morphology to a plate-like shape, which was beneficial to molecular diffusion among its micropores to access the Ti active sites. Therefore, TS-1 synthesized with PAA showed much higher catalytic activity than that synthesized using the traditional synthesis without polyelectrolytes, and only a 30% usage amount of organic template was used. Moreover, this ultra-cheap catalyst also displayed a better catalytic activity than commercial TS-1 synthesized *via* a series of complicated preparation processes including alkene epoxidation with hydrogen peroxide as a green oxidant.

## Abstract 2

Anionic polyelectrolytes can help to directly achieve low-cost, titanium-rich, diffusion restriction-free and 100%-yield titanium silicalite-1.

## Graphical abstract

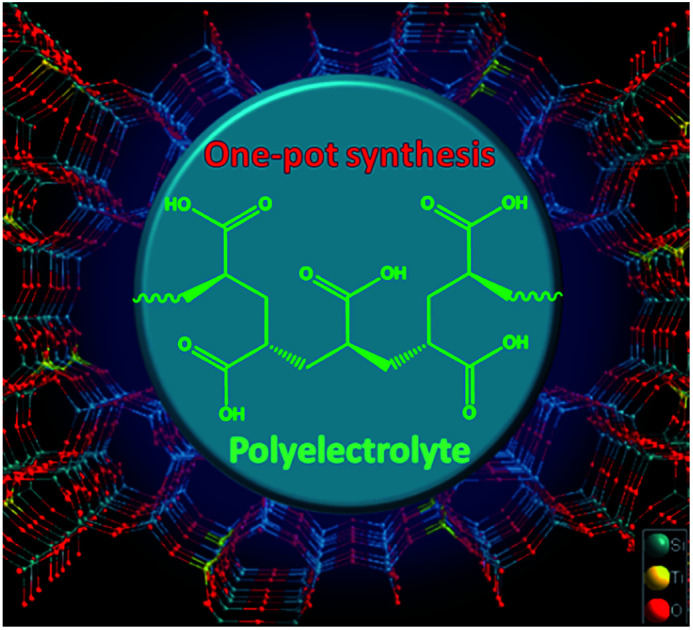

## Introduction

As a successful typical material used in the eco-chemical industry, titanium silicate-1 (TS-1) receives much attention and is very valuable because of its unique catalytic performance combined with H_2_O_2_ as an oxidant. TS-1 was proven to simultaneously realize environmental and economic profit in alkene epoxidation,^1–8^ ketone ammoximation^9,10^ and aromatics hydroxylation.^11^ Furthermore, TS-1 has also shown high catalytic performance in alkane oxidation,^12^ oxidation desulfurization^13,14^ and so on.^15–18^ However, due to the very harsh preparation process and extremely expensive raw materials, the production cost of TS-1 is very high, which has hindered its widespread application. So, reducing the production cost has been a hot research area since the first discovery of this milestone catalyst.

TS-1 was first synthesized in 1983 using tetrapropylammonium hydroxide (TPAOH) as an organic template, tetraethyl orthosilicate (TEOS) as a silicon source and tetraethyl orthotitanate (TEOT) as a titanium source.^19^ This method is called the traditional synthetic method. However, this method requires a large amount of highly pure TPAOH that is strictly free of alkali metal ions, which is the main reason for the high cost of TS-1. Actually, in the traditional synthesis, the contribution of the TPAOH template was more than 96% of the total cost of the raw materials. Although many researchers chose other cheap templates to reduce the production cost, including tetrapropylammonium bromide (TPABr)^7,20–26^ with the same cation TPA^+^ as TPAOH or a mixture of tetraethylammonium chloride (TEACl) and tetrabutylammonium chloride (TBACl),^27^ the catalytic performance was not satisfactory due to the low number of active Ti centers and the large TS-1 crystal size. Therefore, many efforts have focused on developing novel strategies to reduce the production cost by decreasing the usage amount of TPAOH to as low as possible, such as temperature-programmed hydrothermal crystallization, controlled multi-hydrolysis processes and solid-phase crystallization.^28–31^ These strategies were proven to significantly reduce the usage of TPAOH. However, they all required multiple-step synthetic processes, which would significantly increase the production cost including extra equipment and energy. Besides, these methods were complicated and required the cautious operation of matching the hydrolysis and crystallization rates of the Si and Ti sources, which caused difficulty in practical production. This greatly decreased the production reproducibility, leading to a further increase in the production cost. Therefore, a facile and direct route aimed at high-performance and low-cost TS-1 using a small amount of expensive TPAOH template is greatly desired.

In our previous study,^32^ we discovered that poly(acrylic acid) could adjust the crystallization kinetics and modify the crystal size of the titanosilicate molecular sieves to achieve a high-activity titanosilicate catalyst. Here, we employed this unique anionic polyelectrolyte in the low-cost synthesis of TS-1 titanosilicate at a low usage of TPAOH by a one-pot synthesis and compared the catalytic performance with TS-1 synthesized using the traditional synthesis method and current commercial TS-1 catalysts synthesized *via* a complicated multiple-step synthesis and post-treatments.

## Experimental

### TS-1 synthesis

Firstly, titanium tetra-*n*-butoxide(TBOT) was added to hydrogen peroxide aqueous solution, a clear yellow solution was obtained after stirring for 30 minutes and then the tetrapropylammonium hydroxide(TPAOH) was added under stirring. Thereafter, tetraethyl orthosilicate (TEOS) as the silicon source was added to the solution and the solution was stirred overnight to fully hydrolyze. Then, the solution was heated to 353 K to remove alcohol produced by hydrolysis. After cooling down, poly(acrylic acid) (PAA) was added under stirring. The molar composition of the mixture was: SiO_2_/TiO_2_/TPAOH/H_2_O/PAA(unit) = 1 : 0.025 : *x* : 30 : *y*. The mixture was transferred into an autoclave and treated at 443 K for 2 days under static conditions. After treatment, the solid was obtained by filtration or centrifugation, dried at 353 K overnight, and calcined at 823 K for 6 h to remove the organic templates. The samples were named TS-1-*x-y*, where *x* and *y* are the amount of TPAOH and PAA added. For example, TS-1-0.15-0.05 means the molar ratio of SiO_2_/TPAOH was 1 : 0.15 and SiO_2_/PAA(unit) was all 1 : 0.05.

### Characterization

Powder X-ray diffraction (PXRD) was carried out using a Bruker Powder D8 Advance diffractometer operated at 40 kV and 40 mA with CuKα radiation (*λ* = 1.5418 Angstrom). Diffuse reflectance ultraviolet-visible (DRUV/vis) spectra were measured on a Shimadzu UV-2450 spectrophotometer at 298 K with BaSO4 as a reference. IR spectra were measured on a Shimadzu IRPrestige-21 spectrometer as KBr pellets. Elemental analyses (Si and Ti) were measured on an inductively coupled plasma optical emission spectrometer (ICP-OES, Perkin Elmer ICP Optima 2000DV). Field-emission scanning electron microscope (SEM) images were recorded on a JEOL JSM-7600F microscope at 5 kV. Transmission electron microscopy (TEM) observations were performed using a JEOL JEM-1400 TEM microscope working at 100 kV. Particle sizes were measured on Malvern Zetasizer Nano ZS90 analyzer.

### Catalytic reactions

The oxidation reactions were performed with catalyst (25 mg), 1-hexene (5 mmol) and H_2_O_2_ (5 mmol) in methanol (5 ml) in a 20 ml glass reactor with a 333 K oil bath with stirring for 2 h. After the reaction, the mixture was analyzed by gas chromatography. The H_2_O_2_ was determined with a standard Ce(SO_4_)_2_ solution (0.1 M).

## Results and discussion

The synthetic composition and porosity of the synthesized TS-1 samples are summarized in Table 1. The starting Si/Ti ratio was fixed at 40, which was the theoretical possible maximal Ti content incorporated into the TS-1 catalysts.^33^ Considering the usage amount of TPAOH in the traditional synthesis was 0.45 in molar ratio to SiO_2_, herein, the amount of TPAOH was gradually reduced to 0.15, which is only about 30% of the usage amount in the traditional synthesis. Meanwhile, PAA was added to adjust the crystallization kinetics to achieve high catalytic activity.^32^ Moreover, assisted by PAA, the yield of TS-1 was almost 100%. The total titanium source in the synthetic gel was presented in the final TS-1, leading to a Si/Ti ratio of approximately 40. The yield of sample TS-1-0.15-0 was only 82% and the Ti content in TS-1-0.15-0 was slightly less than that of the samples synthesized with PAA. Compared to the traditional synthetic method where TPAOH/SiO_2_ = 0.45, a yield of about 70% and an Si/Ti ratio of 51 could be achieved (TS-1-0.45-0 sample).

**Table 1:** Synthetic composition and porosity of the synthesized TS-1 samples

Samples	Gel composition			Final composition of the TS-1 samples			
	Si/Ti (mol mol^−1^)	TPAOH/SiO_2_ (mol mol^−1^)	PAA/SiO_2_ (mol mol^−1^)	Si/Ti (mol mol^−1^)	*S*_BET_ a(m^2^ g^−1^)	*S*_external_ b (m^2^ g^−1^)	*V*_micro._ (cm^3^ g^−1^)^c^
TS-1-0.15-0.15	40	0.15	0.15	39	401	5	0.19
TS-1-0.15-0.10	40	0.15	0.10	42	386	20	0.17
TS-1-0.15-0.05	40	0.15	0.05	41	433	54	0.18
TS-1-0.15-0	40	0.15	0	46	411	58	0.16
TS-1-0.45-0	40	0.45	0	51	414	74	0.15

a Brunauer–Emmett–Teller (BET) surface area estimated by nitrogen adsorption/desorption measurements.

b External surface area calculated by *t*-plot curves.

c Micropore volume.

**Fig. 1:**
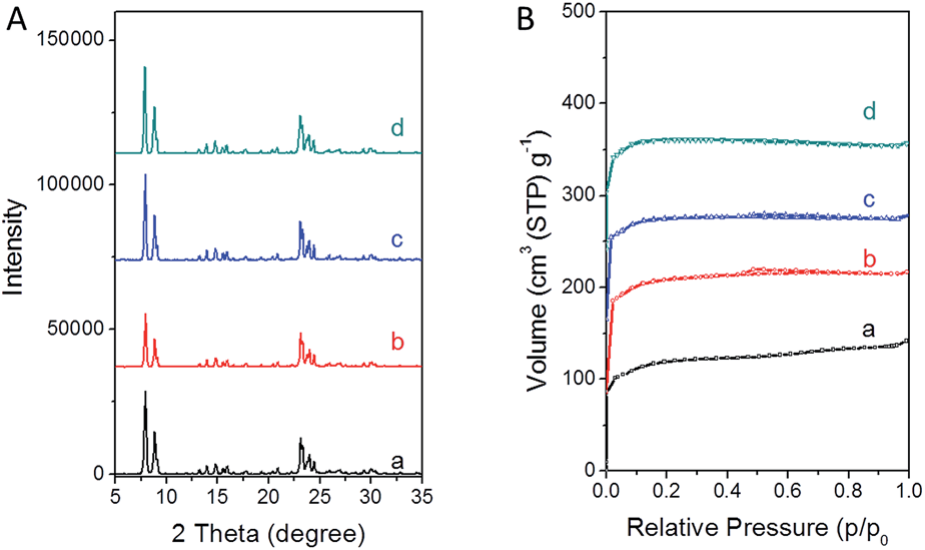
PXRD patterns and nitrogen adsorption/desorption isotherms of the final TS-1 products (a) TS-1-0.15-0, (b) TS-1-0.15-0.05, (c) TS-1-0.15-0.10, and (d) TS-1-0.15-0.15.

The nitrogen adsorption characterization (Fig. 1B) showed all the samples had type I adsorption–desorption isotherms, indicating a microporous structure. In addition, more micropores (volume) were present in TS-1 synthesized with PAA than that from the traditional synthesis (Table 1). The external surface area has a strong relationship with the crystal size. The larger the external surface area is, the smaller the crystal size. Usually, for a microporous catalyst, small-sized crystals possess short microporous channels, which show better catalytic performance due to the easy diffusion of reactants among these short micropores. Comparing TS-1-0.15-0.15, TS-1-0.15-0.10 and TS-1-0.15-0.05, sample TS-1-0.15-0.05 showed the highest external surface area among the samples synthesized with PAA, implying that is has the smallest crystal size. Sample TS-1-0.15-0 synthesized without PAA showed slightly higher external surface area than that of sample TS-1-0.15-0.5, indicating the smaller size of TS-1-0.15-0. It should be noted that sample TS-1-0.45-0 from the traditional synthetic method using a large amount of TPAOH had the largest external surface area among all the TS-1 samples here, indicating the smallest crystal size.

Fig. 1A shows the PXRD patterns of TS-1 synthesized with different amounts of the TPAOH template and the PAA polyelectrolyte. All the samples show characteristic peaks of the MFI-type structure. No other excess peaks were observed, indicating the pure crystallization phase. The low diffraction intensity of sample TS-1-0.15-0.05 was probably due to the noncrystalline raw materials and/or the small crystal size.

**Fig. 2:**
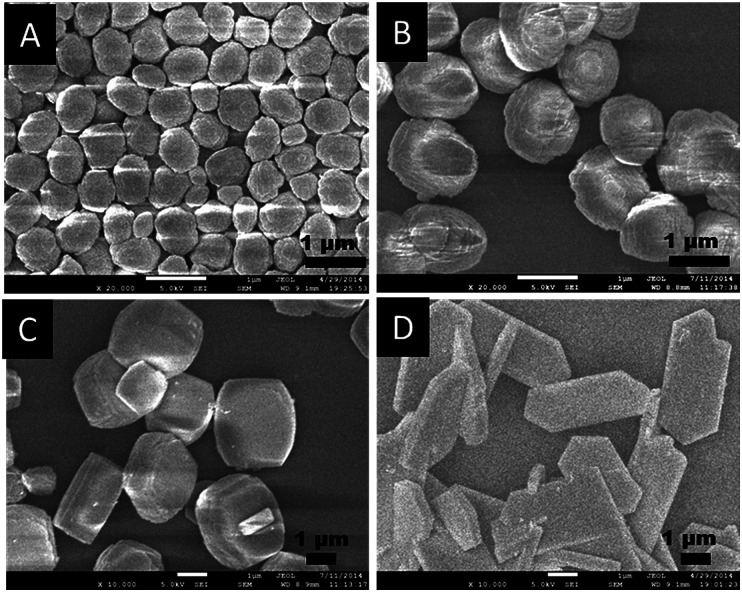
SEM images of the final TS-1 products (A) TS-1-0.15-0, (B) TS-1-0.15-0.05, (C) TS-1-0.15-0.10, and (D) TS-1-0.15-0.15.

**Fig. 3:**
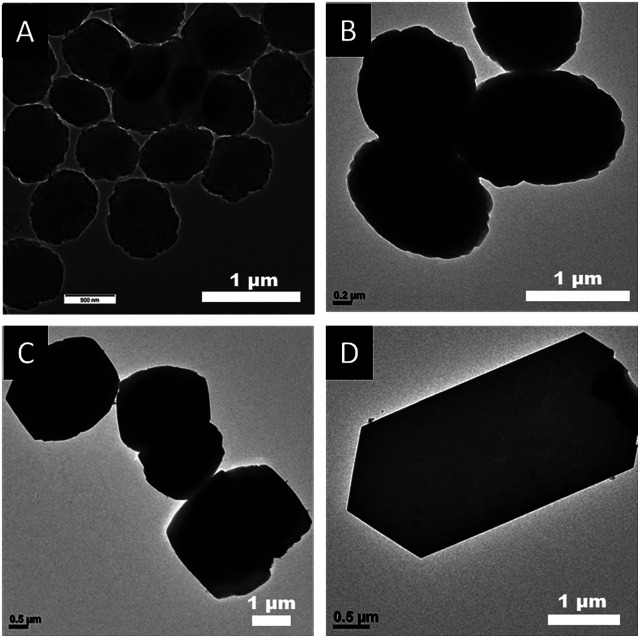
TEM images of final the TS-1 products (A) TS-1-0.15-0, (B) TS-1-0.15-0.05, (C) TS-1-0.15-0.10, and (D) TS-1-0.15-0.15.

As shown in Fig. 2 and Fig. 3, the SEM and TEM images show that TS-1-0.15-0 synthesized without PAA has an ellipsoidal shape with a dimeter of about 0.6 μm. With the increase in the usage amount of PAA, the crystal size of the obtained TS-1 samples increased gradually. Significantly, the morphology changed from spherical to plate-like with uniform thickness. The spherical TS-1-0.15-0.05 sample with a diameter of about 1 micrometer was obtained, which was larger than TS-1-0.15-0. The plate-like sample of TS-1-0.15-0.10 (3.0 × 2.5 × 1.0 μm) had a uniform thickness of about 1 μm. Further increasing the amount of PAA, the plate-like sample of TS-1-0.15-0.15 (5.0 × 2.5 × 0.6 μm) displayed thinner thickness than that of TS-1-0.15-0.10. This characteristic implies that these TS-1 products are still potential high-performance catalysts although they have a large size in the other two dimensions.^34^ It should be noted that TS-1-0.45-0 synthesized by traditional methods had the smallest particle size (100–200 nm) among these TS-1 samples, indicating the traditional method is good at producing small sized TS-1 catalysts.

**Fig. 4:**
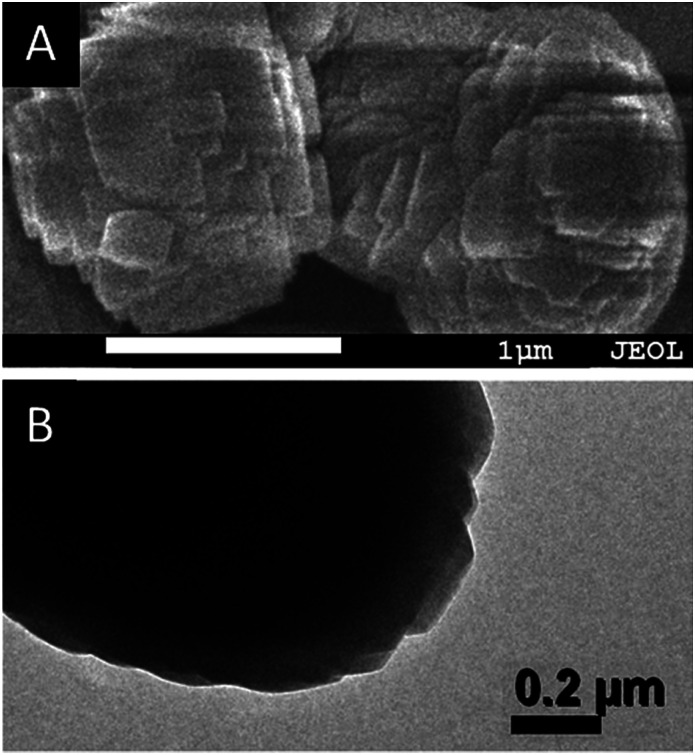
The enlarged (A) SEM and (B) TEM images of TS-1-0.15-0.05.

Interestingly, as shown in the enlarged SEM and TEM images (Fig. 4), the TS-1-0.15-0.05 sample had a rough surface morphology comprised of merged nano-crystals. This may further contribute to its external surface area and further decrease the mass transportation restriction. In addition, the SEM image in Fig. 2B and the TEM image in Fig. 3B revealed that TS-1-0.15-0.05 was completely crystallized without any amorphous raw materials, which suggests that the low intensity in the above PXRD pattern was probably due to these nano-crystals.

For microporous titanosilicates, besides the molecular diffusion restriction in the micropores, the Ti content and state are also important factors for the catalytic reaction. It has been found that there are several different states of Ti species in titanosilicate, including tetracoordinated, hexacoordinated, oligomeric Ti species and small-sized TiO_2_ particles. However, only isolated tetra-coordinated Ti species, namely framework Ti species, have been proven to provide the catalytic activity.^19,35–38^ The coordination state of the Ti species in TS-1 can be detected by DRUV/Vis and FTIR spectroscopy.

**Fig. 5:**
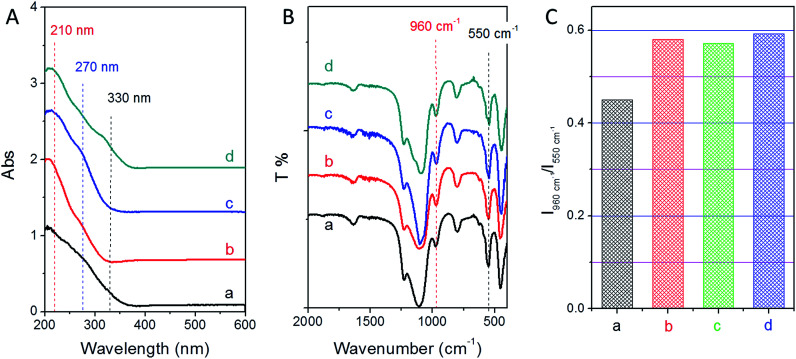
(A) DRUV-vis spectra, (B) FTIR spectra and (C) intensity ratio of 960 cm-1 to 550 cm-1 of the final TS-1 products (a) TS-1-0.15-0, (b) TS-1-0.15-0.05, (c) TS-1-0.15-0.10, and (d) TS-1-0.15-0.15.

As shown in Fig. 5A, all the samples show a main peak at 210 nm, indicating that Ti species are present in the framework. There is a shoulder peak at 270 nm and a small peak at around 330 nm for the sample of TS-1-0.15-0 synthesized without PAA, suggesting that there are significant extra-framework titanium species in this sample. For the samples synthesized with PAA, TS-1-0.15-0.10 shows a shoulder peak at around 270 nm related to the hexacoordinated Ti species. TS-1-0.15-0.15 shows another band at about 330 nm besides 270 nm, indicating the formation of hexacoordinated Ti species and anatase-like TiO_2_ tiny particles. Importantly, TS-1-0.15-0.05 almost shows a peak at 210 nm related to the framework Ti species. Considering a near 100% yield was achieved assisted by PAA and a maximal possible titanium content in the starting gel (Si/Ti molar ratio of 40), it was exciting to find that the sample of TS-1-0.15-0.05 directly achieved the maximal framework titanium content without requiring an excess amount of titanium starting sources. In addition, TS-1 synthesized by the traditional method displayed a slightly lower intensity band at 210 nm as compared to TS-1 assisted by PAA, suggesting a low amount framework titanium species.

The framework titanium in TS-1 titanosilicate could also be observed by the FTIR technique. A band at 960 cm^−1^ in the FTIR spectrum is usually an indication of isomorphous substitution of Ti in the TS-1 lattice. The intensity of this band increases proportionally with an increase in framework Ti content. As shown in Fig. 5B, all the samples show the band at 960 cm^−1^, giving further evidence of framework Ti in all the samples. The intensity of this band was similar for all the TS-1 samples synthesized with the assistance of PAA, which is higher than that of TS-1-0.15-0 synthesized using the traditional method (Fig. 5C). This indicates that PAA can help to incorporate more framework Ti species into TS-1 zeolites. However, the effect of PAA did not directly help Ti to insert into the framework. The mechanism was based on the adjustment of the crystallization mechanism as previous reported.^32^ As a gelating agent, PAA could turn the starting solution to a solid gel, which could result in a solid-phase transformation from the liquid-phase mechanism. It was proven that the best conditions to reach the highest Ti content without extra-framework Ti species was the synergistic effect of the solid-phase and the liquid-phase transformation. Therefore, the optimal amount of PAA should form a mixture composed of both liquid-phase and solid-phase precursors. Here, the sample of TS-1-0.15-0.05 achieved the optimal addition amount of PAA. The samples of TS-1-0.15-0.10 and TS-1-0.15-0.15 also had a higher content of framework Ti species than that of sample TS-1-0.15-0, although some extra-framework and/or anatase-like Ti species were also present.

In addition, a band at 550 cm^−1^ in the FTIR spectrum (Fig. 5B) is characteristic of the vibration of the five-membered ring of MFI structure. All the samples show the band at 550 cm^−1^, suggesting that all the samples had MFI type structures. This result was consistent with the XRD results.

The epoxidation of alkenes is a greatly important industrial process for producing epoxides. Here, the catalytic oxidation of 1-hexene in the presence of H_2_O_2_ was chosen to evaluate the catalytic performance of the synthesized TS-1 catalysts and their catalytic performance was compared with TS-1 from the traditional synthesis method and current commercial TS-1 catalysts.

The catalytic results are summarized in Table 2. All the TS-1 samples assisted by PAA showed better catalytic performance than the TS-1-0.15-0 sample synthesized by the traditional synthesis. Although the amount of TPAOH to silica was increased to 0.45, the TS-1-0.45-0 sample still showed lower catalytic activity than that of TS-1 synthesized with PAA. Here, the crystal size of the TS-1 samples from the traditional synthesis was smaller than that of those synthesized with PAA. The lower catalytic performance of TS-1 from the traditional synthesis may be attributed to the lower framework Ti content as discussed above. The lower turnover numbers (TON) of TS-1-0.15-0 and TS-1-0.45-0 also implied some of the Ti species in the samples were inert for catalytic performance.

**Table 2:** Catalytic oxidation of 1-hexene using various TS-1 catalysts^a^

Samples	Si/Ti (mol mol^−1^)	Crystal size (μm)	Conv. (%)	*S*_epoxide_ b (%)	*E*_H_2_O_2__ (%)^c^	TON^d^ (mol mol^−1^–Ti)
TS-1-0.15-0	46	∼0.6 spherical	9.1	93.1	71	52
TS-1-0.15-0.05	41	∼1.0 spherical	26.5	93.4	84	135
TS-1-0.15-0.10	42	3.0 × 2.5 × 1.0	22.8	93.8	86	118
TS-1-0.15-0.15	39	5.0 × 2.5 × 0.6	24.3	94.6	94	118
TS-1-0.45-0	51	0.1 × 0.1 × 0.1	12.7	97.2	69	80
TS-1^e^	45	∼0.3 spherical	24.4	90.8	80.2	136

a Reaction conditions: catalyst (25 mg), methanol (5 ml), 1-hexene (5 mmol), and H_2_O_2_ (5 mmol), 60 °C, 2 h.

b Selectivity of the epoxide.

c Utilization efficiency of H_2_O_2_ toward the oxidation of 1-hexene.

d Turnover number per Ti site.

e Commercial TS-1 from the Catalysis Society of Japan.

It has been reported that there will be no obvious diffusion restriction when a crystal size less than 1 μm is used for 1-hexene oxidation.^39^ Here, comparing plate-like TS-1-0.15-0.15 (5.0 × 2.5 × 0.6 μm) and plate-like TS-1-0.15-0.10 (3.0 × 2.5 × 1.0 μm), they showed similar 1-hexene conversion and similar turnover numbers (TON) despite different crystal sizes and thicknesses, which supports the above-mentioned conclusion. In addition, assisted by PAA, TS-1 crystals less than 1 μm in length in one dimension (plate-like) or three dimensions (spherical) could be obtained. It was found that 2-dimensional plate-like crystals (TS-1-0.15-0.10 and TS-1-0.15-0.15) showed slightly lower catalytic conversion of 1-hexene and lower TON than spherical crystals with a small size in 3 dimensions (TS-1-0.15-0.05), indicating lower diffusion restriction of spherical small crystals. Meanwhile, it is implied that a high-activity TS-1 catalyst could be achieved if the diffusion length of the crystal was reduced to less than 1 μm in one dimension.

It should be noted that TS-1-0.45-0 from the traditional synthesis showed lower 1-hexene conversion although it had a small size (0.1 × 0.1 × 0.1 μm as shown in Fig. S1†). It would be very easy to transport reactants in such a small crystal. The reason for the low conversion is the low number of active Ti centers as indicated by the low TON, which may be the intrinsic disadvantage of the traditional synthetic system. For commercial TS-1 catalysts with ∼300 nm sized crystals (Fig. S1†), complicated post-treatment processes were still required to improve the catalytic ability of the Ti centers.^40–42^ The TON of commercial TS-1 could reach 136, which is much higher than the TON (80) of TS-1 directly synthesized using the traditional system. However, post-treatment would leach the Ti species from the TS-1 framework, leading to a lower framework Ti content. Comparing TS-1-0.15-0.05 and commercial TS-1, both had a similar TON, indicating the similar catalytic activity of each Ti species. However, TS-1-0.15-0.05 showed a slightly higher conversion of 1-hexene attributed to the higher Ti content. In addition, complicated post-treatment would further increase the production cost due to the usage of new agents and excess energy. Therefore, it was really significant to directly achieve an ultra-low-cost TS-1 catalyst with a high Ti content, high Ti activity and a high yield, which showed great commercial potential.

## Conclusions

We have successfully synthesized a series of low-cost TS-1 titanosilicate molecular sieves by cutting the usage of tetrapropylammonium hydroxide (TPAOH) and assisted with polyelectrolyte PAA. Plate-like and spherical crystals were obtained *via* adjusting the amount of PAA. High-performance TS-1 could still be obtained when the usage amount of TPAOH was reduced by about 70%. PAA played important roles in the synthesis: (1) to change the crystal morphology to achieve less diffusion restriction in one or more dimensions; (2) to directly achieve the maximal framework Ti content (2.0 wt%); (3) to achieve a near 100% yield. The obtained TS-1 catalysts assisted by PAA showed much higher catalytic activity (TON = 118–135) than that from the traditional synthesis (TON = 80). TS-1 from one-pot synthesis assisted with PAA without any other post-treatment steps still showed slightly higher catalytic activity than commercial TS-1 from a series of complicated preparation processes, suggesting promising commercial potential.

## Conflicts of interest

There are no conflicts to declare.

## Supplementary Material

RA-008-C8RA02621A-s001

## References

[cit1] Clerici M. G., Bellussi G., Romano U. (1991). J. Catal..

[cit2] Kumar S. B., Mirajkar S. P., Pais G. C. G., Kumar P., Kumar R. (1995). J. Catal..

[cit3] Serrano D., Sanz R., Pizarroa P., Morenoa I. (2009). Chem. Commun..

[cit4] Wang J., Xu L., Zhang K., Peng H., Wu H., Jiang J.-g., Liu Y., Wu P. (2012). J. Catal..

[cit5] Wang L., Sun J., Meng X., Zhang W., Zhang J., Pan S., Shen Z., Xiao F. S. (2014). Chem. Commun..

[cit6] Kim J., Chunab J., Ryoo R. (2015). Chem. Commun..

[cit7] Zuo Y., Liu M., Zhang T., Meng C., Guo X., Song C. (2015). ChemCatChem.

[cit8] Lin M., Xia C., Zhu B. L., Shu X. (2016). Chem. Eng. J..

[cit9] Le Bars J., Dakka J., Sheldon R. A. (1995). Appl. Catal., A.

[cit10] Zhang T., Wang Y., Wang S., Wu X., Yao P., Lin Y., Xu J. (2015). React. Kinet., Mech. Catal..

[cit11] Lin M. , Shu X. T., Wang X. Q. and Zhu B., China patent, 99126289.1, 1999

[cit12] Huybrechts D. R. C., DeBruycker L., Jacobs P. A. (1990). Nature.

[cit13] Du S., Chen X., Sun Q., Wang N., Jia M., Valtchev V., Yu J. (2016). Chem. Commun..

[cit14] Du S., Li F., Sun Q., Wang N., Jia M., Yu J. (2016). Chem. Commun..

[cit15] Zhou J., Hua Z., Cui X., Ye Z., Cui F., Shi J. (2010). Chem. Commun..

[cit16] Zuo Y., Song W., Dai C., He Y., Wang M., Wang X., Guo X. (2013). Appl. Catal., A.

[cit17] Wróblewska A., Makuch E., Miądlicki P. (2016). Catal. Today.

[cit18] Grieneisen J. L., Kessler H., Fachea E., Govic A. M. L. (2000). Microporous Mesoporous Mater..

[cit19] Taramasso M. , Perego G. and Notari B., *US Pat.*, US4410501, 1983

[cit20] Müller U., Steck W. (1994). Stud. Surf. Sci. Catal..

[cit21] Li G., Wang X., Yan H., Chen Y., Su Q. (2001). Appl. Catal., A.

[cit22] Wang X., Guo X., Li G. (2002). Catal. Today.

[cit23] Wang M., Zhou J., Mao G., Zheng X. (2012). Ind. Eng. Chem. Res..

[cit24] Iwasaki T., Isaka M., Nakamura H., Yasuda M., Watano S. (2012). Microporous Mesoporous Mater..

[cit25] Zhao Q., Hau X., Liu X., Zhai R., Liu L., Bao X., Guo X., Li G., Wang X. (2000). Mater. Chem. Phys..

[cit26] Chen P., Chen X., Chen X., Kita H. (2009). J. Mater. Sci..

[cit27] Xia Q. H., Gao Z. (1997). Mater. Chem. Phys..

[cit28] Deng X., Wang Y., Shen L., Wu H., Liu Y., He M. (2013). Ind. Eng. Chem. Res..

[cit29] Huang D.-G., Zhang X., Liu T.-W., Huang C., Chen B.-H., Luo C.-W., Ruckenstein E., Chao Z.-S. (2013). Ind. Eng. Chem. Res..

[cit30] Ding Y., Ke Q., Liu T., Wang W., He M., Yang K., Jin H., Wang S., Tang T. (2014). Ind. Eng. Chem. Res..

[cit31] Zhang J. H., Yue M. B., Wang X. N., Qin D. (2015). Microporous Mesoporous Mater..

[cit32] Wang J., Zhao Y., Yokoi T., Kondo J. N., Tatsum T. (2014). ChemCatChem.

[cit33] Millini R., Previde Massara E., Perego G., Bellussi G. (1992). J. Catal..

[cit34] Fu K., Yao J., Xiao Q., Liu H., Li T., Tatsumi T., Wang J.-G. (2016). RSC Adv..

[cit35] Fan W., Duan R.-G., Yokoi T., Wu P., Kubota Y., Tatsumi T. (2008). J. Am. Chem. Soc..

[cit36] Guo Q., Sun K., Feng Z., Li G., Guo M., Fan F., Li C. (2012). Chem.–Eur. J..

[cit37] Bordiga S., Damin A., Berlier G., Bonino F., Ricchiardi G., Zecchina A., Lamberti C. (2001). Int. J. Mol. Sci..

[cit38] Wang J.-G., Wang Y., Tatsumi T., Zhao Y. (2106). J. Catal..

[cit39] Wendland M. S., Zimmerman S. C. (1999). J. Am. Chem. Soc..

[cit40] Tsai S.-T., Chao P.-Y., Tsai T.-C., Wang I., Liu X., Guo X.-W. (2009). Catal. Today.

[cit41] Wu X., Wang Y., Zhang T., Wang S., Yao P., Feng W., Lin Y., Xu J. (2014). Catal. Commun..

[cit42] Hammond C., Tarantino G. (2015). Catalysts.

