# Supplementary material for: Anionic polyelectrolytes in titanosilicate molecular sieve synthesis towards simultaneously accomplishing low production cost and high catalytic activity
Source: RSC Adv. 2018 Jun 13;8(38):21363–8. doi: 10.1039/c8ra02621a (PMC9080955; doi:10.1039/c8ra02621a)
Supplement: RA-008-C8RA02621A-s001 [file RA-008-C8RA02621A-s001.pdf]

Electronic Supplementary Material (ESI) for RSC Advances.
This journal is © The Royal Society of Chemistry 2018

Supporting information for:

Anionic polyelectrolytes in titanosilicate molecular sieve synthesis towards simultaneously accomplishing low production cost and high catalytic activity

Kairui Fu,[a] Jingui Wang,*[a] Yichen Wang,[a] Yuanchao Shao,[a] Jiaqi Zhu,[a] and Tianduo Li*[a]

[a] Shandong Provincial Key Laboratory of Fine Chemicals, School of Chemistry and Pharmaceutical Engineering, Qilu University of Technology (Shandong Academy of Sciences), Jinan 250353 P.R. China.

E-mail: JGWang@qlu.edu.cn; ylpt6296@vip.163.com

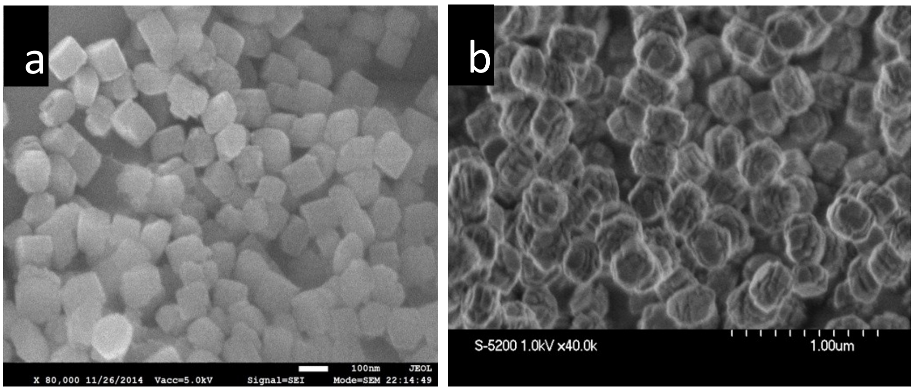

Fig. S1 SEM images of (a) TS-1-0.45-0, (b) commercial TS-1.
